# Coral carbon isotope sensitivity to growth rate and water depth with paleo-sea level implications

**DOI:** 10.1038/s41467-019-10054-x

**Published:** 2019-05-03

**Authors:** Braddock K. Linsley, Robert B. Dunbar, Emilie P. Dassié, Neil Tangri, Henry C. Wu, Logan D. Brenner, Gerard M. Wellington

**Affiliations:** 1 Lamont-Doherty Earth Observatory of Columbia University, 61 Route 9W, Palisades, NY 10964 USA; 2 Department of Environmental Earth Systems Science, Stanford University, Stanford, CA 94305 USA; 3 CNRS, EPOC, UMR 5805, Place du Dr B. Peyneau, F-33120 Arcachon, France; 4 Leibniz Centre for Tropical Marine Research (ZMT) GmbH, Fahrenheitstraße 6, 28359 Bremen, Germany; 5 Department of Biology, University of Houston, Houston, TX 77204 USA

**Keywords:** Biogeochemistry, Palaeoceanography, Stable isotope analysis, Marine biology

## Abstract

Although reef coral skeletal carbon isotopes (δ^13^C) are routinely measured, interpretation remains controversial. Here we show results of a consistent inverse relationship between coral δ^13^C and skeletal extension rate over the last several centuries in *Porites* corals at Fiji, Tonga, Rarotonga and American Samoa in the southwest Pacific. Beginning in the 1950s, this relationship breaks down as the atmospheric ^13^C Suess effect shifts skeletal δ^13^C > 1.0‰ lower. We also compiled coral δ^13^C from a global array of sites and find that mean coral δ^13^C decreases by −1.4‰ for every 5 m increase in water depth (*R* = 0.68, *p* < 0.01). This highlights the fundamental sensitivity of coral δ^13^C to endosymbiotic photosynthesis. Collectively, these results suggest that photosynthetic rate largely determines mean coral δ^13^C while changes in extension rate and metabolic effects over time modulate skeletal δ^13^C around this mean value. The newly quantified coral δ^13^C-water depth relationship may be an effective tool for improving the precision of paleo-sea level reconstruction using corals.

## Abstract (web summary)

Rising anthropogenic CO_2_ levels in the atmosphere are resulting in ocean acidification which may impact coral growth rates. Here, the authors quantify the relationship between water depth and δ^13^C compositions of South Pacific corals from the pre-industrial era, and their results should lead to improvements in the precision of sea level reconstructions using fossil corals.

## Introduction

**Fig. 1:**
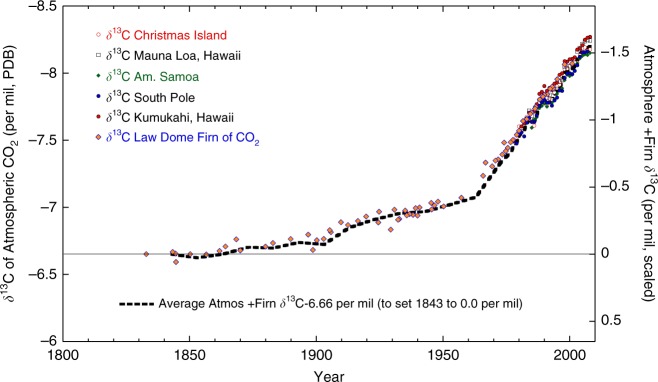
The atmospheric ^13^C Suess effect: atmospheric CO_2_ δ^13^C results from stations at Christmas Island, Mauna Loa, Kumukahi, American Samoa, and the South Pole^41^ compared to Antarctic firn CO_2_ δ^13^C ^2^. Black bold dashed line is our calculated average of these results that represents the ^13^C Suess effect. We subtracted this average from our coral δ^13^C results from Fiji, Tonga, Rarotonga, and American Samoa to remove the ^13^C Suess effect from these coral δ^13^C series

Hermatypic (i.e., reef building) corals face an uncertain future due to the accelerating input of anthropogenic CO_2_ into the surface ocean and from rising ocean temperatures. Excess atmospheric CO_2_ is largely derived from fossil fuel combustion and deforestation. This has led to a decline of atmospheric ^13^C/^12^C ratio (δ^13^C) of CO_2_ synchronous with the rise of atmospheric CO_2_ concentrations^1,2^. Decreasing atmospheric δ^13^C has also affected surface ocean dissolved inorganic carbon (DIC) δ^13^C via atmosphere–ocean exchange of CO_2_ and is recognized as the oceanic ^13^C Suess effect^3^. The Suess effect accelerated beginning around 1960 CE in conjunction with more rapidly rising atmospheric CO_2_ levels (Fig. 1). This influx of excess atmospheric CO_2_ could negatively impact coral calcification as surface ocean pH declines^4^. Reef corals are increasingly bleaching—expelling their colorful endosymbiotic zooxanthellae when the coral animal is stressed, typically due to the sustained exceedance of local water temperature thresholds. The frequency of widespread bleaching events appears to have increased from 3 reported major bleaching events in the 1876–1979 period to more than 60 during 1979–1990^5^ and now to reports of significant bleaching at some locations every year since 1990^6,7^. Given these stressors on reef corals, there is a need to identify geochemical tracers of the coral calcification process to reconstruct and better understand changes in coral growth processes in the past to develop a baseline against which to assess future changes.

Hermatypic coral skeletal δ^13^C reflects changes in coral calcification processes as well as surface ocean DIC δ^13^C. However, the origin of changes in coral δ^13^C on different time-scales is controversial due to complex interactions between physiological and chemical processes that involve strong isotopic fractionation^8–13^. The most widely accepted view is that coral skeletal δ^13^C is controlled by a combination of metabolic mechanisms related to photosynthesis and respiration^11^, kinetic effects related to calcification rate^8,14^ and possibly seawater pH^15,16^.

Kinetic fractionation results from discrimination against the heavy isotopes of both carbon and oxygen during the hydration and hydroxylation of CO_2_ at the site of calcification^8,9,14,17,18^. The relatively slow exchange of oxygen and carbon isotopes between dissolved CO_3_^2−^ and seawater compared to the fast rate of calcification is thought to be the main mechanism preventing δ^18^O and δ^13^C equilibrium during CaCO_3_ precipitation^8,14,16^. The expected result is that rapid skeletal growth is associated with lower skeletal δ^13^C^17,19,20^.

Metabolic fractionation is thought to produce additional changes in skeletal δ^13^C, likely arising from changes in the δ^13^C of the DIC pool at the site of calcification^8,14,21^. Two primary processes that may change the interstitial carbon pool are autotrophy (photosynthesis and respiration) and heterotrophic prey capture^12,17,18,22–25^. Photosynthesis by endosymbiotic zooxanthellae affects coral skeletal δ^13^C by preferentially consuming ^12^CO_2_, resulting in ^13^C enrichment in the DIC in the internal calcification pool^9,14^. As the rate of photosynthesis increases and/or the rate of respiration decreases, the internal carbon pool used by the coral animal to make its skeleton becomes relatively enriched in ^13^C resulting in higher skeletal δ^13^C^8,11,12,18^. Intensity of photosynthesis depends on several environmental factors such as water depth and transparency, solar irradiance levels, cloud cover, and sea surface temperature (SST)^8,11,19,23,26^. Heterotrophy by corals introduces nutritional resources to the coral polyp that are typically depleted in ^13^C (δ^13^C ranging from −14 to −25‰ and lower).

Coral skeletal δ^13^C values can also be directly related to changes in zooxanthellae density within the coral^27–29^. Rising water temperatures that surpass habitable thresholds can reduce zooxanthellae density in coral tissues short of complete bleaching^30^, lowering the photosynthetic uptake of ^12^C and decreasing skeletal δ^13^C. Porter et al.^29^ calculated that a loss of ~80% of the symbiotic zooxanthellae will lead to a decline of 1‰ in coral δ^13^C.

Many studies have interpreted 20th century trends towards lower coral skeletal δ^13^C to be the result of the ^13^C Suess effect in the surface ocean^9,10,13,31–37^. The magnitude and timing of this coral δ^13^C trend varies between sites and not all corals exhibit δ^13^C trends. For example, Swart et al.^10^ evaluated trends toward lower δ^13^C in multiple globally-distributed corals and sclerosponges. Of the 37 corals included in that study, 23 (62%) had a statistically significant trend toward lower δ^13^C from 1900 to the present leading to the conclusion that this trend was related to the ^13^C Suess effect. A multi-coral composite δ^13^C time series from Fiji generated using *Porites* coral cores has a long-term decreasing trend of ~1.5‰ over a 221-year period interpreted to be due to the ^13^C Suess effect^13^. The Fijian corals also exhibit a moderate correlation between skeletal linear extension rate and annual average skeletal δ^13^C (5 coral average *R* = −0.37) with a mid-20th century increase in the trend toward lower skeletal δ^13^C while the respective skeletal extension rates remained generally constant.

In this report, we show that annually averaged coral skeletal δ^13^C is inversely correlated with skeletal extension rate in five multi-century long coral δ^13^C time series from Fiji, Tonga, Rarotonga, and American Samoa in the South Pacific. This relationship breaks down however in the mid-1900s when the atmospheric ^13^C Suess effect disrupts the relationship between skeletal extension and δ^13^C. We also have compiled coral δ^13^C from a global array of sites where water depth to the top of the coral is known and find a robust inverse relationship between mean coral δ^13^C and water depth. This highlights the fundamental sensitivity of coral δ^13^C to endosymbiotic photosynthesis. We discuss how this relationship can potentially be used to improve the precision of paleo-sea level reconstructions by providing better constraint on the growth position water depth of individual fossil corals.

## Results

### South Pacific *Porites* coral δ^13^C and extension rates

**Fig. 2:**
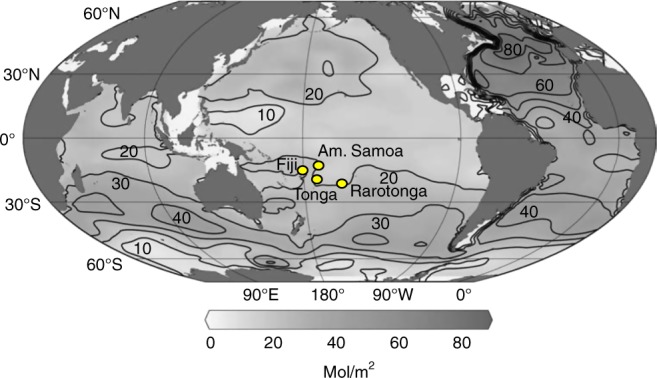
Map of anthropogenic CO_2_ inventory in the global ocean^59^ and locations of our South Pacific study sites: location of our coral extension rate and skeletal δ^13^C time series from Fiji, Tonga, Rarotonga, and American Samoa in relationship to anthropogenic CO_2_ water column inventory (mol m^−2^) in the Pacific. Approximately 20 mol m^−2^ of CO_2_ has been taken up by the upper ocean at each of these study sites

**Fig. 3:**
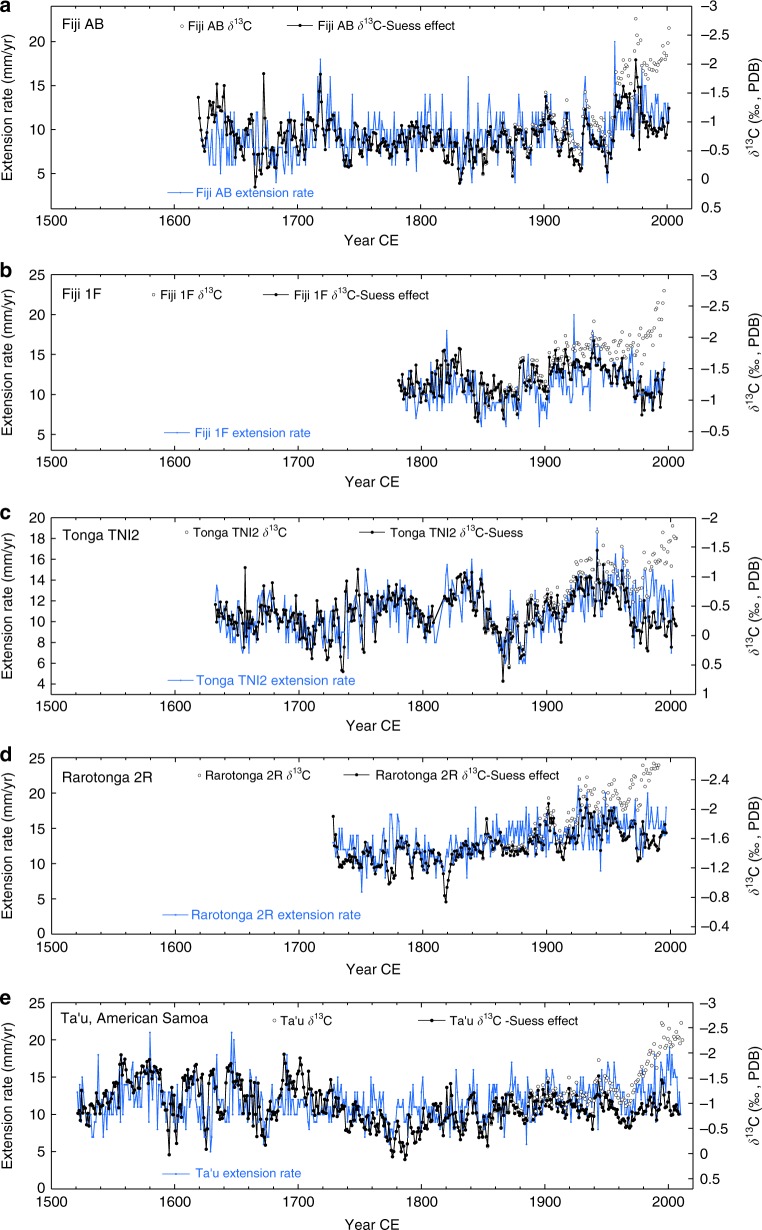
South Pacific coral δ^13^C and skeletal extension rate: coral annual skeletal extensions rates (in blue) and annual average skeletal δ^13^C (black open dots) and δ^13^C data with the average atmospheric ^13^C Suess effect removed (solid black dots) in *Porites lutea* corals from: **a** Fiji core AB^53^, **b** Fiji core 1F^52^, **c** Tonga core TNI2^54^, **d** Rarotonga core 2R^51,52^, and **e** American Samoa core Ta’u-1^55,56^. Extension rate and δ^13^C significantly correlate to varying degrees (see *R* values in Table 1) up until the mid-20th century when coral δ^13^C abruptly shifts ~1.0‰ lower at all of these sites. The very low growth year in 2000 in Tonga core TNI2 is a confirmed bleaching event when growth was truncated to 7 mm in that year. Also note that Fiji cores 1F and AB were collected from *Porites lutea* colonies in Savusavu Bay at the same water depth approximately 100 m from each other. Source data are provided as a Source Data file in the Supplement

Building on the results of Dassié et al.^13^, we examined the relationship between annual extension rates and annually averaged coral skeletal δ^13^C in multi-century long *Porites* coral cores from Tonga, Rarotonga, and American Samoa for comparison to Fiji (Figs. 2 and 3) (see analytical methods and note that the two sampled Fijian corals that were sampled are located 100 m from each other at the same water depth in Savusavu Bay). We calculated annual skeletal extension rates in four of the corals (Fiji core 1F, Fiji core AB, Rarotonga core 2R, and American Samoa core Ta’u-1) using the age-models developed from sub-annual resolution coral δ^18^O results. For Tonga core TNI2 we measured annual extension rates by hand from X-ray positive images because this coral had clear annual density bands and skeletal δ^18^O and δ^13^C had been generated on annually averaged samples.

**Fig. 4:**
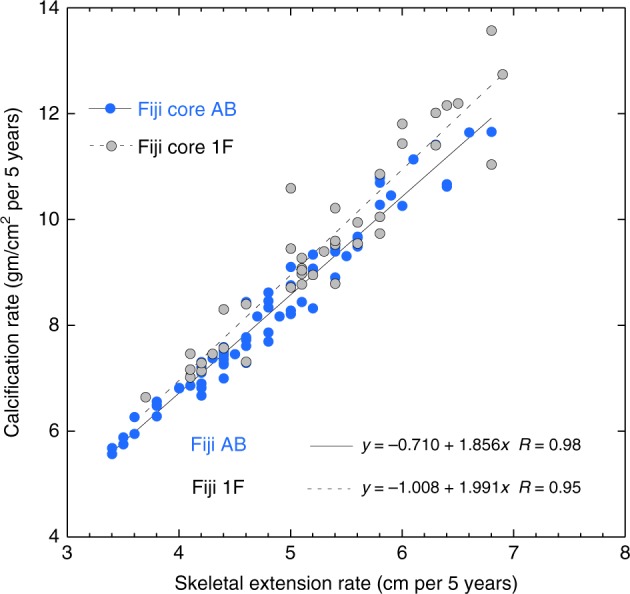
Fiji *Porites* coral extension and calcification rates: correlation of skeletal extension rate and calcification rate in Fiji coral cores 1F and AB. Data calculated and presented as 5-year averages of extension and calcification. Correlation coefficients (*R* values) for core 1F = 0.95 and for core AB = 0.98. Source data are provided as a Source Data file in the Supplement

*Porites* annual skeletal extension rates have been shown to be highly correlated to calcification rates^38–40^ indicating that linear extension can be a proxy for calcification rate. For two of our cores from Fiji we have calculated calcification rate in 5-year non-overlapping intervals from density determined from computerized tomography (CT) scans and extension rate (see Methods and Fig. 4). The correlation coefficient (*R*) between extension rate and calcification rate is 0.95 for Fiji 1F and 0.98 for Fiji AB (*p* < 0.0005). This indicates that, at least for the Fiji cores, extension rate can be used as a direct proxy for calcification. Given the relatively homogeneous character of the *Porites* cores and slabs from Tonga, Rarotonga, and Ta’u, we assume a relatively uniform skeletal density and therefore the same strong correlation between extension rate and calcification rate for the other 3 cores shown in Fig. 3.

The comparison of coral extension rates and annual skeletal δ^13^C at Fiji, Tonga, Rarotonga, and American Samoa shows these two parameters are moderately and consistently inversely correlated over the last several hundred years until the mid-20th century with faster skeletal extension correlating with lower annual average δ^13^C (see Fig. 3 and Table 1). Faster coral growth discriminates against the heavy carbon isotope (^13^C). We observe insignificant correlations between skeletal δ^13^C, extension rates, and SSTs (Supplementary Table S1). In four of the coral δ^13^C records, decadal-scale 0.5–1.0‰ increases in skeletal δ^13^C prior to 1960 are associated with 50% reductions in growth rate at various times in the past several centuries. This suggests that extension rate alone can generate 0.5–1.0‰ deviations in coral δ^13^C from the mean value. An additional important observation is that prior to the mid-20th century, changes in extension rate and skeletal δ^13^C are not synchronous between sites. This indicates that skeletal δ^13^C was primarily controlled at each site by local metabolic and kinetic calcification processes and not related to the changes in the external carbon pool. Although the exact proportion of metabolic vs. kinetic effects in each coral cannot be determined, the correlation between lower extension rate and higher δ^13^C in conjunction with local colony-specific skeletal δ^13^C and extension rate relationships points to extension rate control on skeletal δ^13^C over much of the last several hundred years until the mid-20th century or some other unknown parameter controlling both.

**Table 1:** Pearson correlation coefficients for Fiji, Tonga, Rarotonga, American Samoa (Ta’u); coral skeletal annual δ^13^C and annual extension rate (in mm)

(A) Correlation coefficients for coral skeletal δ^13^C and extension rate							
Fiji, Tonga, Rarotonga, Am. Samoa (Ta’u); Annual coral extension rate and annual skeletal δ^13^C (all *Porites lutea*)							
		*R* all data	*p*	*R* Before 1960	*p*	*R* After 1960	*p*
Fiji 1F		−0.45	<0.001	−0.55	<0.001	−0.33	<0.01
Fiji AB		−0.47	<0.001	−0.34	<0.001	−0.12	<0.01
Tonga TNI2		−0.64	<0.001	−0.68	<0.001	−0.3	<0.01
Rarotonga 2R		−0.55	<0.001	−0.55	<0.001	−0.32	<0.01
Ta’u-1		−0.39	<0.001	−0.33	<0.001	−0.38	<0.01
	**Average** ***R***	−**0.5**		−**0.49**		−**0.29**	
**(B) Correlation coefficients for coral skeletal δ** ^**13**^ **C and extension rate (3-year smoothed)**							
		*R* All data 3 pt	*p*	*R* Before 1960	*p*	*R* After 1960	*p*
Fiji 1F		−0.59	<0.001	−0.71	<0.001	−0.01	<0.01
Fiji AB		−0.64	<0.001	−0.47	<0.001	−0.14	<0.01
Tonga TNI2		−0.77	<0.001	−0.80	<0.001	−0.28	<0.01
Rarotonga 2R		−0.71	<0.001	−0.70	<0.001	−0.22	<0.01
Ta’u-1		−0.57	<0.001	−0.50	<0.001	−0.69	<0.01
	**Average** ***R***	−**0.65**		−**0.64**		−**0.27**	
**With** ^**13**^ **C Suess effect subtracted (see Methods)**							
**(C) Correlation coefficients for coral skeletal δ** ^**13**^ **C-Suess effect and extension rate**							
		*R* All data	*p*	*R* Before 1960	*p*	*R* After 1960	*p*
Fiji 1F		−0.42	<0.001	−0.45	<0.001	−0.58	<0.01
Fiji AB		−0.39	<0.001	−0.32	<0.001	−0.02	<0.01
Tonga TNI2		−0.58	<0.001	−0.66	<0.001	−0.47	<0.01
Rarotonga 2R		−0.49	<0.001	−0.49	<0.001	−0.17	<0.01
Ta’u-1		−0.28	<0.001	−0.30	<0.001	−0.45	<0.01
	**Average** ***R***	−**0.43**		−**0.44**		−**0.34**	
**(D) Correlation coefficients for coral skeletal δ** ^**13**^ **C-Suess effect and extension rate (3-year smoothed)**							
		*R* All data 3 pt	*p*	*R* Before 1960	*p*	*R* After 1960	*p*
Fiji 1F		−0.61	<0.001	−0.66	<0.001	−0.77	<0.01
Fiji AB		−0.54	<0.001	−0.45	<0.001	−0.1	<0.01
Tonga TNI2		−0.71	<0.001	−0.79	<0.001	−0.64	<0.01
Rarotonga 2R		−0.64	<0.001	−0.65	<0.001	−0.2	<0.01
Ta’u-1		−0.4	<0.001	−0.44	<0.001	−0.62	<0.01
	**Average** ***R***	−**0.58**		−**0.60**		−**0.47**	

(A) *R* values for coral δ^13^C and extension rate for all data, data before 1960, and data after 1960. (B) Same as in (A), except on 3-year running averaged versions of coral δ^13^C and extension rate. (C) *R* values for coral δ^13^C with ^13^C Suess effect removed and extension rate for all data, data prior to 1960 and data after 1960. (D) Same as in (C), except on 3-year running averaged versions of coral δ^13^C minus ^13^C Suess effect and extension rate

Beginning in the mid-to-late 1900s, coral δ^13^C shifts significantly lower at all five sites while coral extension rates do not significantly increase or decrease. The timing of the δ^13^C shift varies somewhat between sites, but all sites recorded large amplitude shifts to >1.0‰ more negative values by the mid-1970s. This results in a lower correlation between coral extension rates and δ^13^C in 4 of the 5 coral records (see Table 1). The decoupling of coral extension rates and skeletal δ^13^C at all five sites in the late 20th century suggests that the ^13^C Suess effect overwhelmed the coral extension rate and skeletal δ^13^C relationship. To test this, we compiled Pacific Island-based atmospheric δ^13^C of CO_2_ data^41^ and Antarctic firn δ^13^C of CO_2_ data^2^ and generated an annually averaged ^13^C Suess effect that we scaled and then subtracted from each coral δ^13^C series (see Fig. 1 and Methods). The resulting coral δ^13^C time series are shown in each panel of Fig. 3 compared to the raw δ^13^C data. Removal of the Suess effect from the coral δ^13^C series highlights the large amplitude of the Suess effect on coral δ^13^C and lowers the overall correlations with extension rate slightly, with some mixed improvement in correlation for the data after 1960 (see Table 1). This exercise suggests that the major control on the decoupling of these coral δ^13^C series and extension rates is the ^13^C Suess effect and not changes in calcification processes related to the ^13^C Suess effect.

### Coral skeletal δ^13^C sensitivity to water depth

**Fig. 5:**
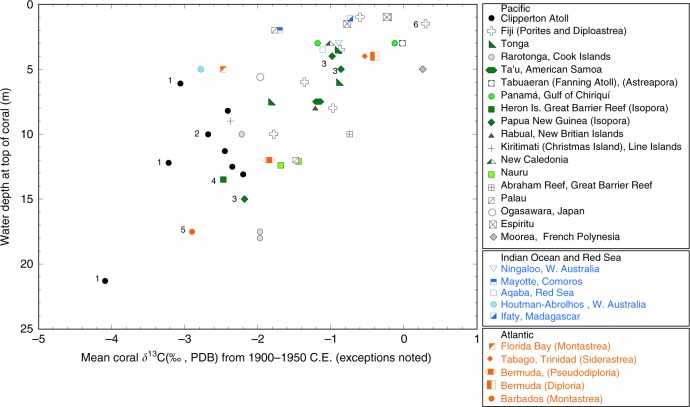
Coral skeletal δ^13^C to water depth comparison: mean coral δ^13^C compared against water depth at top of coral for different locations and coral genera based on our own records and a literature search compilation. Most sites are in the Pacific. Those corals that are not *Porites* are indicated in legend. Where possible, mean coral δ^13^C was calculated over the period from 1900 to 1950 to avoid the interval of most pronounced ^13^C Suess effect because the magnitude of the ^13^C Suess effect varies with region. Sites with shorter records after 1950 are indicated with numbers (1 = Clipperton 1990–1994; 2 = Clipperton *Pavona* (1946–1958); 3 = Papua New Guinea *Isopora* (1970s); 4 = Heron Is. *Isopora* (2008–2012); 5 = Barbados *Montastrea* (1976–1978)). The black open cross numbered 6 is a Fiji *Diploastrea*. Despite the different coral genera and wide spatial array of sites, the results indicate a decrease in coral δ^13^C with growth position water depth of ~1.4‰ per 5 m of depth (*R* = 0.67, *p* < 0.01) (see Supplementary Fig. 1). If all the corals with δ^13^C data younger than 1950 are removed the relationship is −1.5‰ per 5 m of depth (*R* = 0.60, *p* < 0.01) (see Supplementary Fig. 2). Source data are provided as a Source Data file in the Supplement. References for coral δ^13^C data are also included in the supplement

To further explore other factors responsible for average coral skeleton δ^13^C at these and other sites, we evaluate coral δ^13^C and growth position water depth (water depth of coral top at time of collection) for different sites and coral genera using data available in the literature and some of our unpublished data (Supplementary Table S2). Other researchers have noted a relationship between water depth and coral δ^13^C ^8,12,13,19,24,26,42,43^. In Fig. 5, we show bulk coral δ^13^C results plotted against water depth where we were able to obtain information about water depth to the top of the coral. To minimize the influence of the ^13^C Suess effect, where possible, we have averaged coral δ^13^C from 1900 to 1950 for comparison to water depth, otherwise we averaged whatever portion of the time series fell within the 1900–1950 interval. This was not possible for several of the shorter coral records younger than 1950 that are noted. There is a statistically significant linear relationship between coral δ^13^C and water depth where coral δ^13^C decreases ~1.4‰ for every 5 m of water depth (*R* = −0.68; *p* < 0.001) (−0.28‰ per 1 m increase) (Fig. S1). If all the corals with δ^13^C data after 1950 are removed the relationship is −1.5‰ per 5 m of depth (*R* = −0.60, *p* < 0.01) (Fig. S2). Coral skeletal δ^13^C values are ~0.0 to −0.8‰ at the shallowest sites (1–2 m) and decrease to −4‰ at 21 m (one Clipperton *Porites* coral currently anchors the deep end of the scale). This comparison supports earlier suggestions that bulk coral skeletal δ^13^C is related to water depth due to rapidly declining solar irradiance with depth (~50% of photosynthetically active radiation at 20 m^26,44,45^) resulting in less photosynthesis and lower δ^13^C in the internal carbon pool used for calcification. In addition, at deeper depths it is possible that the fraction of carbon derived from heterotrophic feeding increases also resulting in lower δ^13^C in skeletons of deeper corals. Site-specific differences in water clarity and/or mean cloud cover may explain some of the between site δ^13^C variability with water depth. Since our comparison contains several coral genera (mostly *Porites*, but also *Isopora*, *Astreapora*, *Diploastrea*, *Diploria*, *Montastraea* (*reclassified as Orbicella*), and *Siderastrea*), this relationship is not *Porites* specific. Although this mean coral δ^13^C vs. water depth relationship only has an *R* of 0.68 suggesting that other factors are involved, we conclude that the water depth vs. coral δ^13^C relationship across multiple sites and coral genera supports the hypothesis that mean coral skeletal δ^13^C is fundamentally related to photosynthetic rate in the coral tissue.

## Discussion

Our South Pacific coral δ^13^C and skeletal extension rate results point to the long-term inverse relationship between extension rate and mean coral δ^13^C until the mid-20th century with faster skeletal extension generally occurring when skeletal δ^13^C is lower. Beginning in the mid-1900s the accelerating atmospheric ^13^C Suess effect acted to progressively decouple the long-term relationship between coral δ^13^C and extension rate as ^13^C depleted CO_2_ was taken up by the surface ocean. Although the atmospheric ^13^C Suess effect forcing of CO_2_ into the surface ocean would have been relatively uniform across our South Pacific study region, the removal of the ^13^C Suess effect from the coral δ^13^C series shows that the response of coral chemistry to this forcing was not uniform. For example, compare the two Fiji cores 1F and AB which were collected at the same water depth, ~100 m from each other in Savusavu Bay, Fiji (Fig. 3). In these two corals, both the pre-1950s and post-1950s variability in δ^13^C and extension rate are different and not correlated. Also compare the δ^13^C results from the two Fiji cores to Tonga core TNI2 and Rarotonga core 2R and American Samoa core Ta’u-1. The local and regional differences between individual coral δ^13^C and extension rate records indicate that, other than the Suess effect, there are colony-specific factors that control decadal changes in coral δ^13^C and extension rate. The fact that extension rate did not decline in these corals as the Suess effect impacted the isotopic composition of the internal carbon pool suggests there have been no obvious negative effects on growth of these *Porites* corals due to the excess CO_2_ in the surface ocean (thus far), a conclusion in-line with other studies of *Porites* corals^46^. However, it remains unclear exactly why the relationship between extension rate and skeletal δ^13^C broke down during the late 20th-century and why large 0.5‰ changes in individual coral δ^13^C accompanied by synchronous changes in extension rate occurred prior to the 1950s.

SST variability in the region does not appear to be a factor effecting either skeletal extension rate or δ^13^C. As mentioned above, our analysis of instrumental SST from all four sites and coral Sr/Ca derived SST for Fiji, Tonga, and Rarotonga indicates no synchronous change in SST that correlates with the decoupling of coral growth rates and skeletal δ^13^C in these corals^47^ and insignificant correlation coefficients (see Supplementary Table S1). 20th century SST in the region displays decadal changes related to the Pacific Decadal Oscillation (PDO) but are not correlated with our observed changes in coral δ^13^C or extension rate.

Decreasing water depth at the top of the coral as the calcifying surface shoals over time also does not appear to be an important factor in the 20th century trends in coral δ^13^C at Fiji, Tonga, Rarotonga, and American Samoa. In the raw coral δ^13^C data, the trend toward lower δ^13^C in the late 1900s is opposite that expected based on the water depth vs. skeletal δ^13^C relationship shown in Fig. 5. The water depth effect on coral δ^13^C we have observed would work to slightly dampen the ^13^C Suess effect trend. The fact the amplitude of the coral δ^13^C trend in the late 1900s is equal to that expected from the atmospheric Suess effect suggests that other factors, such as shoaling water depth as corals accrete vertically had little influence on the late 20th century declining coral δ^13^C at these South Pacific sites. When the ^13^C Suess effect is removed the detrended δ^13^C series display some significant decadal-scale changes that do not correlate between sites and also with inconsistent long-term trends (see Fig. 3). We conclude that the effect of declining water depth as the corals living surface grows upwards has only a small effect on these coral δ^13^C secular trends.

In the absence of other obvious environmental parameters driving coupled coral skeletal δ^13^C and extension rate variability prior to the mid-20th century, one possibility is that changes in heterotrophy was influential for both skeletal δ^13^C and extension rate. Although this factor cannot be substantiated here, a greater proportion of coral carbon derived from zooplankton and other detritus would be predicted to lower skeletal δ^13^C^12,17,18,22–25^. It is possible that coral zooplankton feeding rates or zooplankton abundance and/or detrital composition in the surface ocean is related to skeletal extension rates. Evaluating this possibility will require additional research.

The water depth influence on coral skeletal δ^13^C discussed earlier and displayed in Fig. 5 indicates that photosynthetic rate has a large influence on determining or setting the mean δ^13^C skeletal value in the upper ~20 m of the water column. Changing growth rate and metabolic effects over time then modulate skeletal δ^13^C around this mean value. This newly quantified coral δ^13^C vs. water depth relationship may have implications for paleo-sea level reconstructions that utilize massive, symbiont-bearing fossil corals. Instead of assuming a growth position water depth for fossil massive coral samples, mean bulk skeletal δ^13^C could be used to constrain the growth position water depth for a particular coral sample. In particular, massive *Porites* and *Pavona* corals are known to grow over a large range of water depths in clear water^45,48,49^. As an example, in the ultra-clear waters surrounding Clipperton Atoll in the eastern North Pacific, *Porites lobata* colonies were found living from 3 to 35 m and *Pavona varians* was found living from 5 m to nearly 50 m water depth^49^. In Hawaii, active reef growth of mostly *Porites  *sp. corals, occurs today down to 50 m^45^. In fossil massive corals collected in drill cores into ancient reefs, often in situ corals that are dated to reconstruct sea level are assumed to have grown near the shallow end of this range^50^. This assumption could be a major source of error in sea level reconstruction studies that utilize massive corals. We suggest that the use of the relationship shown in Fig. 5 and Supplementary Fig. 1 could constrain growth depths of fossil corals to within 7 m of their true in situ depth (±3.5 m). An age limitation for this approach arises due to the possibility that atmospheric CO_2_ δ^13^C and surface ocean DIC δ^13^C has varied over time. Thus, generating relative water depth estimates based on mean coral δ^13^C values from corals of vastly different ages could be biased by differences in background DIC δ^13^C if the atmospheric CO_2_ concentration was significantly different. Although additional research is needed to better understand the water depth and mean coral δ^13^C relationship shown in Fig. 5, utilizing skeletal δ^13^C to constrain growth position depth would be an improvement over the current approach of just assuming water depth based on modern coral ecology and relying on core depth measurements of recovered fossil corals.

Down-core annually averaged *Porites* sp. coral δ^13^C values from Fiji, Tonga, Rarotonga, and American Samoa in the South Pacific are shown to be inversely correlated with annual skeletal extension rates over the last several hundred years up until the mid-20th century. Temporal variability in extension rates and coral δ^13^C appears to be coral colony-specific and even differs between colonies at the same location. Beginning in the mid-1900s, coral δ^13^C shifts >1.0‰ lower at all four sites due to the anthropogenic ^13^C Suess effect and δ^13^C correlation with extension rate declines. To remove the influence of the Suess effect from the coral δ^13^C series,  we combined instrumental atmospheric CO_2_ δ^13^C and Antarctic firn CO_2_ δ^13^C to create a composite average ^13^C Suess effect. We then subtracted this composite from each coral δ^13^C record. The resulting detrended δ^13^C series reveal that coral extension rates appear to be unaffected by the late 1900s increased influx of CO_2_ into the surface ocean. To evaluate other effects on coral skeletal δ^13^C, we have also compiled mean coral δ^13^C and growth position water depth data from throughout the tropics with focus on the Pacific. This compilation reveals a significant trend towards lower skeletal δ^13^C as depth increases (−1.4‰ per 5 m of depth; *R* = 0.68, *p* < 0.01). This observation highlights the fundamental sensitivity of coral δ^13^C to endosymbiotic photosynthesis and may have utility in constraining the paleo water depth of in situ fossils corals (±3.5 m) thereby improving sea level reconstructions. Combined, these results suggest that coral growth rates and water depth are two key factors influencing skeletal δ^13^C. As more coral time series δ^13^C results are made available, the relationships we discuss can be refined to improve the utility of coral δ^13^C as a tracer of coral growth processes and changing environmental conditions.

## Methods

### Rarotonga core 2R

Core information, analytical methods, and chronology development for core 2R have been previously discussed^51^. In summary, the 3.6 m core from a *Porites lutea* colony was collected in 18 m of water depth on the SW side of Rarotonga in April 1997. 1 mm interval samples from 1 to 845 mm (1997–1943) and every-other 1 mm samples from 846 to 3659 mm were analyzed for δ^13^C. 11% of the samples were analyzed in replicate with an average difference of 0.06‰. The standard deviation of the NBS-19 standards analyzed daily was 0.04‰.

### Fiji cores 1F and AB

Fiji cores 1F (water depth to coral top = 10 m) and AB (water depth to coral top = 8 m) were collected from different *P.  lutea* coral colonies in April 1997 and December 2001, respectively in Savusavu Bay on the north island of Vanua Levu Fiji. The two coral colonies were located approximately 100 m apart. Procedures for the isotopic analyses have been previously discussed^51–54^. For the first 14 years of growth, every 1 mm sample (200 samples) was analyzed, and below this depth every other 1 mm sample was analyzed. Analysis of every other 1 mm sample resulted in a resolution of about 6–7 samples per year. Replicate samples were analyzed every 8 samples (every 16 samples after 200 mm). The average difference between duplicate δ^13^C analyses was 0.066‰. Samples of international standard NBS-19 were interspersed every ~10 samples. The standard deviation of NBS-19 standards analyzed for δ^13^C was ±0.016‰.

### Tonga core TNI2

In November 2004 at the Tonga Island of Nomuka Iki (20°16′ S; 174° 49′ W) two useable cores were collected from a large living colony of *P. lutea* in 3.5 m of water (TNI2-H1, 4.03 m useable length; and TNI2-H3, 1.8 m useable length)^54^. Because of a bio-eroded zone from ~1 m to 1.68 m in TNI2-H1, TNI2-H3 was drilled to allow sampling around the bio-eroded zone by splicing the δ^18^O and δ^13^C records from core H3 onto core H1. A total of 2195 samples were analyzed in TNI2. Details of the isotopic analysis methods and chronology development for Tonga core TNI2 were previously presented^54^. The average difference between the 138 replicate δ^13^C measurements in TNI2 was 0.06‰.

### American Samoa core Ta’u-1

In November 2011, we cored a large colony of *P.  lutea* on the western side of the island of Ta’u located at S 14° 15′ 33.74″: W 169° 30′ 01.61″ (or S 14 15.566, W 169 30.027) on an exposed outer reef in 7.5 m of water (water depth to top of coral)^55,56^. Prior to slabbing, the longest core, Tau-1, was scanned by X-ray computer automated tomography (CT) to reveal growth bands and to determine the optimal cutting planes (CT collage of coral cores in Tangri et al.^56^). The core was then slabbed on a two-bladed tile saw to produce 5 mm thick slabs. The last section (41 cm) of the core was recovered from the drill barrel as rubble; in order to slab and sample this section, we epoxied the pieces together with West Marine 6-10 epoxy. We then chose overlapping transects which avoided epoxy and visible bioerosion.

We sampled the slabs using a 35 rpm Dremel tool and 1.5 mm drill bit at ~1 mm intervals, producing 6476 samples, including transect overlaps but not replicates. The top half of the core was analyzed at Lamont-Doherty Earth Observatory (LDEO) on either an Elementar Isoprime mass spectrometer equipped with a dual-inlet and Multiprep or a Themo-Fisher Delta V+ mass spectrometer with dual-inlet and Kiel IV carbonate reaction device. The instruments are in the same laboratory at LDEO and have been cross-calibrated. They use the same CO_2_ reference gas and dewatered phosphoric acid is made using the same protocols for each instrument. With the Isoprime we dissolved ~80–120 µg coral powder aliquots in ~100% H_3_PO_4_ at ~90 °C. With the Delta V-Kiel IV we dissolved ~50–80 µg coral powders in ~100% H_3_PO_4_ at ~70 °C. NBS-19 standards were analyzed 5–6 times per day. To assess external precision and sample homogeneity, 209 replicate samples were analyzed (8.2% replication). The standard deviation of NBS-19 standards analyzed was 0.03‰ for δ^13^C. The average difference of the 209 replicate δ^13^C analyses was 0.082‰. The bottom half of the core was analyzed at Stanford University on a Finnigan MAT 252 coupled to a Kiel III carbonate autosampler, which added 3 drops of ~100% H_3_PO_4_ at ~70 °C to each sample of ~75 µg prior to analysis. Samples were corrected to NBS-19 and MS2, an in-house standard. Internal consistency of the standards was 0.065‰ (δ^18^O) and 0.033‰ (δ^13^C), and 244 (7.6%) of the samples were replicated. The average difference of the replicates was 0.052‰ (δ^18^O) and 0.069 (δ^13^C). All results are reported relative to VPDB (in ‰).

The samples from the core’s live-collected top serve to anchor the chronology for the δ^18^O series to November 2011. Below this section, annual δ^18^O minima and maximum were attributed to seasonal maxima and minima in SST, respectively. Verification of this approach comes from pseudo-coral forward modeling where we used instrumental SST and sea surface salinity (SSS) to generate a modeled coral δ^18^O series from 1981 to 2008^55^. The final chronology for Ta’u-1 based annual banding and annual δ^18^O cycles extends from 1521 to 2011 CE with an average extension rate of 1.2 cm yr^−1^. The core preparation and isotopic analytical methods of the Ta’u-1 analyses were previously published^55,56^.

### ^13^C Suess effect removal from coral δ^13^C series

To remove the ^13^C Suess effect for our South Pacific coral δ^13^C records we elected to compile and average annual resolution atmospheric δ^13^C of CO_2_ results from the Pacific Islands and South Pole spanning 1978–2008^41^. We then merged this composite average with the Antarctic firn δ^13^C of CO_2_ data from Law Dome cores DEO8 and DE08-2^2^ (see Fig. 1). Only cores DEO8 and DEO8-2 were selected of the Law Dome data since these firn cores had the best-constrained age models and CO_2_ lock-in depth when the pore space CO_2_ was isolated from the atmosphere^2^. Since the firn data was unevenly spaced in time and had associated age model error and possible variability in lock-in depth, we first generated 10-year averages of the firn results spanning the length of these cores with reported dates from 1843 to 1993. Then we interpolated the 10-year averages into 1-year increments and merged this with the atmospheric CO_2_ results. The approach created a smoothed version of the atmospheric δ^13^C of CO_2_ change which we believe is more representative of background secular changes in δ^13^C of CO_2_ at our study sites in Fiji, Tonga, Rarotonga, and American Samoa. We scaled this δ^13^C of CO_2_ data such that subtraction from our coral δ^13^C series resulted in 0.00 per mil correction in the 1840s (see Fig. 1). The average atmospheric δ^13^C of CO_2_ data shown in Fig. 1 we argue represents secular changes in background conditions. We realize that local surface ocean conditions at each site can influence the uptake of atmospheric CO_2_, thus there could be local processes that influence the magnitude of the Suess effect. However, we believe that our approach effectively removed the background, secular trend in atmospheric CO_2_ δ^13^C termed the Suess effect.

### Calcification rate in Fiji cores 1F and AB

For Fiji cores 1F and AB (see Figs. 2 and 3 and above in Methods) 7 mm thick coral slabs were scanned for skeletal density on a medical X-ray CT scanner^57,58^. CT scans were acquired at the Albany Advanced Imaging Center in Albany, New York, using a General Electric LightSpeed RT-16 (16 slice X-ray CT scanner). All samples were sent through the imaging chamber with scanning parameters between 120 and 150 keV and at 200 mA. Linear attenuation coefficients measured by CT scanners were not in conventional density units but in a standardized CT scale known as Hounsfield units. The units are linearly correlated to bulk density calibration standards of known wet bulk density to form a calibration curve allowing for the conversion of coral skeletal CT measurements into density units of g cm^−3^, ref. ^58^. Calibration measurements and the derived calibration curve for the CT scanner were made from density standards of precision cut rectangular blocks to calculate dry and bulk weight density. The standards were made from a variety of massive, mounding, hermatypic coral specimens with skeletons spanning a range of densities along with other various CaCO_3_ samples (solid aragonite and calcite minerals). After calibration, average skeletal density (g cm^−3^) was measured at 5-year increments following the chronology determined by each cores age model developed from δ^18^O and Sr/Ca data. At each 5-year marker affixed to the coral slab surface, density was measured in an area approximately equivalent to ±2 years of skeletal growth surrounding the time marker. This density value was assumed constant in that 5-year increment and multiplied by the total skeletal extension in that 5 years of growth to determine the average calcification rate for the particular 5-year section of coral in g cm^−2^ year^−5^. Figure 4 shows the strong linear relationship between calcification rate and extension rate in both Fiji cores (Fiji 1F, *R* = 0.95; Fiji AB, *R* = 0.98).

## Supplementary information

Supplementary Information

Source data

## Data Availability

The calculated average atmospheric CO_2_ δ^13^C data displayed in Fig. 1 and the coral data presented in Figs. 3–5 are included as a supplementary data file (Source data.xlsx) and are also archived at https://www.ncdc.noaa.gov/data-access/paleoclimatology-data. Requests for data presented in this contribution can also be addressed to B.K.L.
