# Author Correction: Coral carbon isotope sensitivity to growth rate and water depth with paleo-sea level implications

**DOI:** 10.1038/s41467-019-11041-y

**Published:** 2019-06-28

**Authors:** Braddock K. Linsley, Robert B. Dunbar, Emilie P. Dassié, Neil Tangri, Henry C. Wu, Logan D. Brenner, Gerard M. Wellington

**Affiliations:** 1 Lamont-Doherty Earth Observatory of Columbia University, 61 Route 9W, Palisades, NY 10964 USA; 2 Department of Environmental Earth Systems Science, Stanford University, Stanford, CA 94305 USA; 3 CNRS, EPOC, UMR 5805, Place du Dr B. Peyneau, F-33120 Arcachon, France; 4 Leibniz Centre for Tropical Marine Research (ZMT) GmbH, Fahrenheitstraße 6, 28359 Bremen, Germany; 5 Department of Biology, University of Houston, Houston, TX 77204 USA

Correction to: *Nature Communications* 10.1038/s41467-019-10054-x, published online 03 May 2019.

The original version of the Source Data associated with this Article included an error, in which the ‘Fiji AB d13C-Suess’ data point and the ‘TNI2 d13C-Suess Effect’ data point for the year ‘1950.5’ where incorrectly omitted from the Figure 3 tab. The missing values are ‘−0.24’ and ‘−0.64’, respectively. The HTML has been updated to include a corrected version of Source Data.

