# Supplementary material for: Coral carbon isotope sensitivity to growth rate and water depth with paleo-sea level implications
Source: Nat Commun. 2019 May 3;10:2056. doi: 10.1038/s41467-019-10054-x (PMC6499886; doi:10.1038/s41467-019-10054-x)
Supplement: Supplementary file 1 — Supplementary Information [file 41467_2019_10054_MOESM1_ESM.pdf]

Supplementary Information

Coral Carbon Isotope Sensitivity to Growth Rate and Water Depth with Paleo-Sea Level Implications

Braddock K. Linsley et al.

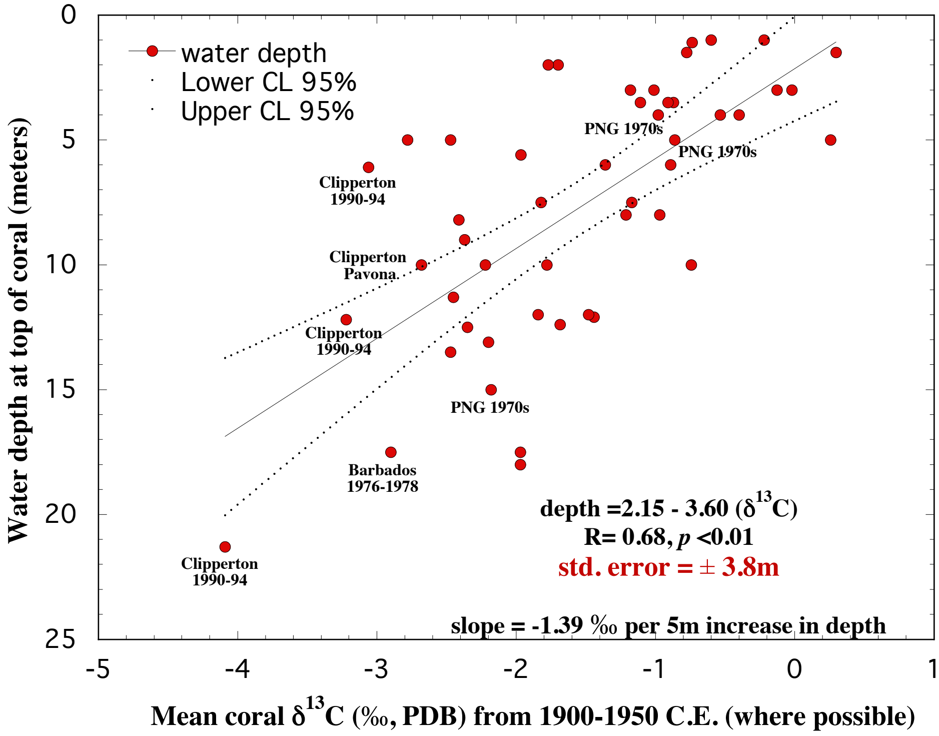

Supplementary Figure 1: Coral skeletal $\delta^{13}C$ vs. water depth at top of coral (same data as displayed in Figure 5). The 95% upper and lower confidence levels (CL) for the slope are indicated. The standard error of the relationship of 3.8m suggests that we can use mean pre-Suess Effect coral $\delta^{13}C$ to potentially constrain the water depth of fossil corals to within 7m of their actual growth position depth. Source data are provided as a Source Data file in the Supplement.

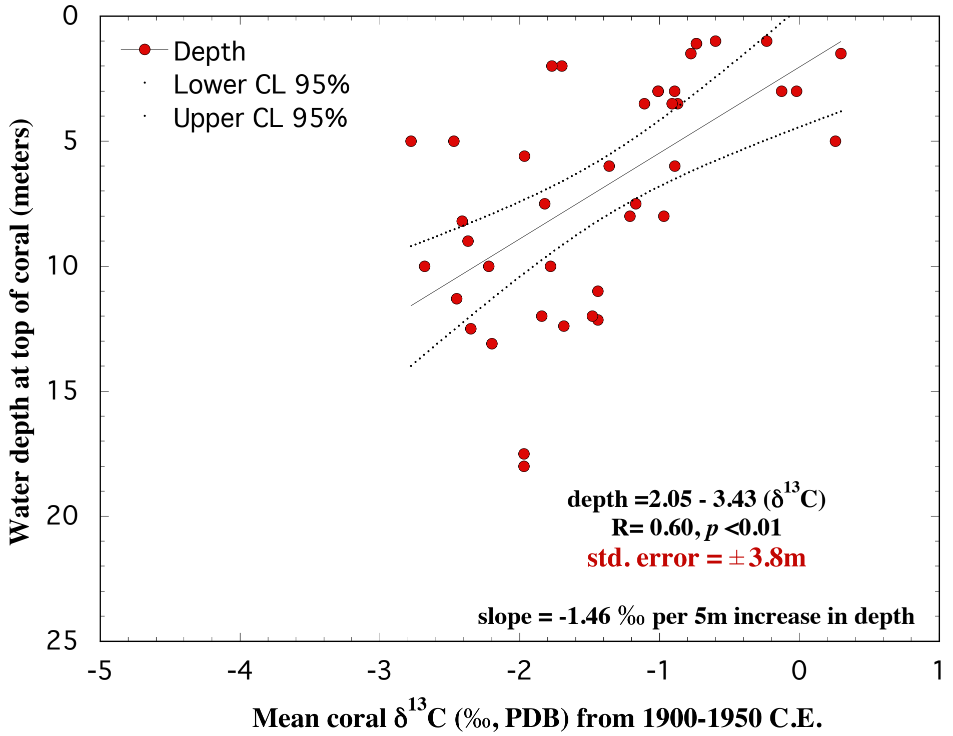

Supplementary Figure 2: Coral skeletal $\delta^{13}C$ vs. water depth at top of coral for all corals with data between 1900 and 1950. All corals younger than 1950 shown in Figures 5 and Supplementary Figure 1 are excluded. The 95% upper and lower confidence levels (CL) for the slope are indicated. The standard error of the relationship of 3.8m suggests that we can use mean pre-Suess Effect coral $\delta^{13}C$ to potentially constrain the water depth of fossil corals to within 7m of their actual growth position depth.

Supplementary Table 1: (A & B) Pearson correlation coefficients for Fiji, Tonga, Rarotonga and American Samoa (Ta'u) coral $\delta^{13}C$ and extension rates to sea surface temperature. Here we evaluated correlations to both ERSST (Rayner et al., 2003 [1]) and coral Sr/Ca derived SST estimates (Linsley et al., 2000 [2] ; Linsley et al., 2004 [3] ; Wu et al., 2013 [4] ; Linsley et al., 2015 [5]) over the period from 1855 to the top of each core in the late 20th century.

(A) Correlation coefficients for coral skeletal $\delta^{13}C$-Suess effect and SST and coral Sr/Ca SST

	R All data ERSST		R All data Sr/Ca SST	
Fiji 1F	0.04	<0.001	-0.21	<0.001
Fiji AB	0.04	<0.001	0.17	<0.001
Tonga TNI2	0.22	<0.001	0.06	<0.001
Rarotonga 2R	0.05	<0.001	0.32	<0.001
Ta'u-1	0.05	<0.001	n.a	<0.001
**Average R**	**0.08**		**0.09**	

(B) Correlation coefficients for coral skeletal extension rates and SST and coral Sr/Ca SST

	R All data ERSST		R All data Sr/Ca SST	
Fiji 1F	0.20	<0.001	0.11	<0.001
Fiji AB	0.06	<0.001	0.25	<0.001
Tonga TNI2	0.00	<0.001	0.29	<0.001
Rarotonga 2R	0.09	<0.001	0.23	<0.001
Ta'u-1	0.00	<0.001	n.a	<0.001
**Average R**	**0.07**		**0.22**	

Supplementary Table 2: Average $\delta^{13}C$ and water depth data plotted in Figure 5. Location, sample ID, coral genera, year interval over which coral $\delta^{13}C$ was averaged, water depth to top of coral, average $\delta^{13}C$ (in ‰ PDB), reference for data.

Location	Core/Sample ID	Genera	Year Interval	water depth (m)*	Average $\delta^{13}C$ in interval	Reference
Clipperton Atoll	C4B	*Porites*	1900-1950	8.2	-2.41	Linsley et al., 2000b [6]
Clipperton Atoll	C2B	*Porites*	1937-1950	13.1	-2.20	Linsley et al., 2000b [6]
Clipperton Atoll	CDT20	*Porites*	1990-1994	6.1	-3.06	this study
Clipperton Atoll	CDT40	*Porites*	1990-1994	12.2	-3.22	this study
Clipperton Atoll	CDT70	*Porites*	1990-1994	21.3	-4.09	this study
Clipperton Atoll	C3C	*Porites*	1900-1950	12.5	-2.35	Linsley et al., 1999 [7]
Clipperton Atoll	6A	*Porites*	1945-1950	11.3	-2.45	Linsley et al., 2000b [6]
Clipperton Atoll	B101	*Pavona*	1946-1958	10	-2.68	this study
Fiji	1F, Savusavu Bay	*Porites*	1900-1950	10	-1.78	Linsley et al. 2004 [3], this study
Fiji	AB, Savusavu Bay	*Porites*	1900-1950	8	-0.97	Linsley et al., 2004 [3], this study
Fiji	FVB1, Vanua Balavu	*Porites*	1900-1950	6	-1.36	Dassie et al., 2013 [8]; 2014 [9]
Fiji	16F, Aiwa Island	*Porites*	1900-1950	3.5	-0.87	Dassie et al., 2013 [8]; 2014 [9]
Fiji	FVB2, Vanua Balavu	*Porites*	1900-1950	1	-0.60	Dassie et al., 2013 [8]; 2014 [9]
Fiji	Diplo LH, Savusavu Bay	*Diploastrea*	1900-1950	1.5	0.30	Bagnato et al., 2004 [10] , this study
Tonga	TF1, Funoifua Island	*Porites*	1900-1950	7.5	-1.82	Linsley et al., 2008 [11], this study
Tonga	TNI2, Nomuka Iki Island	*Porites*	1900-1950	3.5	-0.91	Linsley et al., 2008 [11] this study
Tonga	TM1, Malinoa Island	*Porites*	1900-1950	6	-0.89	Linsley et al., 2008 [11], this study
Rarotonga	2R, west side	*Porites*	1900-1950	18	-1.97	Ren et al., 2002 [12], Linsley et al., 2004 [3]; this study
Rarotonga	99, west side	*Porites*	1906-1950	17.5	-1.97	Linsley et al., 2006

						[13]; this study
Rarotonga	3R, north side	*Porites*	1900-1950	10	-2.22	Linsley et al., 2006 [13]; this study
Am. Samoa	Tau-1, Ta'u Island	*Porites*	1900-1950	7.5	-1.17	Linsley et al., 2017 [14] Tangri et al.,2018 [15]; this study
Fanning Atoll	FII Astreopora, east side	*Astreapora*	1900-1950	3	-0.02	Stolorow 2006 [16]; this study
Gulf of Chiriquí, Panamá	Secas Island	*Porites*	1900-1950	3	-0.13	Linsley et al., 1994 [17]; Brenner et al., 2016 [18]; this study
Gulf of Chiriquí, Panamá	Coiba Island	*Porites*	1900-1950	3	-1.18	Brenner et al., 2016 [18]; this study
Heron Is., Great Barrier Reef	Her13	*Isopora*	2008-2012	13.5	-2.47	Brenner et al., 2017 [19]; this study
Papua New Guinea	PNG0036	*Isopora*	1970s	4	-0.98	Lemley 2012 [20]; this study
Papua New Guinea	PNG0089	*Isopora*	1970s	5	-0.86	Lemley 2012 [20]; this study
Papua New Guinea	PNG0021A	*Isopora*	1970s	15	-2.18	Lemley 2012 [20]; this study
Rabaul, W. Pacific	Rabaul	*Porites*	1900-1950	8	-1.21	Quinn et al., 2006 [21]
Christmas Is., Line Islands	Christmas Is.	*Porites*	1900-1950	9	-2.37	Evans et al., 1999 [22]
New Caledonia	New Caledonia	*Porites*	1900-1950	3	-1.01	Quinn et al., 1998 [23]
Nauru	Nauru	*Porites*	1900-1950	12.1	-1.44	Guilderson and Schrag 1999 [24]
Nauru	Nauru	*Porites*	1900-1950	12.4	-1.68	Guilderson and Schrag [24]
Great Barrier Reef	Abraham Reef	*Porites*	1900-1950	10	-0.74	Druffel and Griffin 1993 [25]; 1999 [26]
Tabago	Tabago	*Sidereastrea*	1932-1952	4	-0.53	Moses and Swart 2006 [26]
Western Australia	Houtmann-Abrolhos	*Porites*	1900-1950	5	-2.78	Kuhert et al., 1999 [28]

Red Sea	Aqaba	*Porites*	1900-1950	3.5	-1.11	Hess et al., 1994 [29]
Madagascar	Mayotte	*Porites*	1900-1950	2	-1.70	Zinke et al 2009 [30]
Madagascar	Ifaty Reef	*Porites*	1900-1950	1.1	-0.73	Zinke et al 2004 [31]
Moorea	Nigaloo Reef	*Porites*	1900-1950	5	0.26	Boiseau et al., 1999 [32]
Japan	Ogasawara	*Porites*	1900-1950	5.6	-1.97	Felis et al 2009 [33]
Palau	Rock Island	*Porites*	1900-1950	12	-1.48	Osborne et al., 2014 [34]
Palau	Rock Island	*Porites*	1900-1950	2	-1.77	Osborne et al., 2014 [34]
Vanuatu	Espiritu Santo	*Porites*	1928-1950	1.5	-0.77	Kilbourne et al., 2004 [35]
Vanuatu	Tangoa Island off Espiritu	*Plarygyra*	1900-1950	1	-0.22	Quinn et al., 1993 [36]
Florida	Florida Bay	*Montastrea*	1900-1950	5	-2.47	Swart et al., 1996 [37]
Bermuda	NE breakers	*Pseudodiploria*	1927-1950	12	-1.84	Kuhnert et al., 2005 [38]
Bermuda	North Rock	*Diploria*	1900-1950	4	-0.40	Nozaki et al., 1978 [39]
Barbados	Barbados	*Montastrea*	1976-1978	17.5	-2.90	Guilderson et al., 2001 [40]

Supplementary References:

1 Rayner, N. A., Parker, D. E. Horton, E. B., Folland, C. K., Alexander. L. V., Rowell, D. P., Kent, E. C. & Kaplan, A. Global analyses of sea surface temperature, sea ice, and night marine air temperature since the late nineteenth century. *J. Geophys. Res*. 108, 4407 (2003).

2 Linsley, B.K., Wellington, G. M. & Schrag, D. P. Decadal Sea Surface Temperature Variability in the Sub-tropical South Pacific from 1726 to 1997 A.D. *Science,* vol. 290, 1145-1148, (2000).

3 Linsley, B.K., Wellington, G. M., Schrag, D. P., Ren, L., Salinger, M. J. & Tudhope, A. W. Coral evidence for changes in the amplitude and spatial pattern of South Pacific interdecadal climate variability over the last 300 years, *Climate Dynamics,* 22: 1-11, doi:10.1007/s00382-003-0364-y, (2004).

4 Wu, H. C., Linsley, B. K., Dassie, E. P., Schiraldi, B. & de Menocal, P. B. Oceanographic variability in the South Pacific Convergence Zone region over the last 210 years from multi-site coral Sr/Ca records, *Geophys. Geochem. Geosys*. 14, no 5, 1435-1453, doi:10.1029/2012GC004293, (2013).

5 Linsley, B. K., Wu, H. C., Dassié, E. P. & Schrag, D. P. Decadal changes in South Pacific sea surface temperatures and the relationship to the Pacific decadal oscillation and upper ocean heat content, *Geophys. Res. Lett.,* 42, doi:10.1002/2015GL063045 (2015).

6 Linsley, B.K., Ren, L., Dunbar, R.B. & Howe, S.S. ENSO and decadal-scale climate variability at 10°N in the Eastern Pacific from 1893 to 1994; A coral-based reconstruction from Clipperton Atoll, *Paleoceanography,* vol. 15, no. 3, 322-335, (2000).

7 Linsley, B.K.; Messier, R.G. & Dunbar, R.B. Assessing between colony oxygen isotope variability in the coral *Porites lobata* at Clipperton Atoll, *Coral Reefs,* vol. 18 (1), 13-27 (1999).

8 Dassié, E.P., Lemley, G. M. & Linsley, B. K. The Suess effect in Fiji coral $\delta^{13}C$ and its potential as a tracer of anthropogenic CO2 uptake, *Palaeogeography, Palaeoclimatology, Palaeoecology*, 370, 30-40, (2013).

9 Dassié, E. P., Linsley, B. K., Correge, T., Wu, H. C., Lemley, G. M., Howe, S. S. & Cabioch, G. A Fiji multi-coral $\delta^{18}O$ composite approach to obtaining a more accurate reconstruction of the last two-centuries, *Paleoceanography*, 18 DEC 2014, DOI: 10.1002/2013PA002591 (2014).

10 Bagnato, S., Linsley, B. K., Howe, S. S., Wellington, G. M. & Salinger, J. Evaluating the use of the massive coral *Diploastrea helipora* for paleoclimate reconstruction, *Paleoceanography*, 19, PA1032, doi:10.1029/2003PA000935, (2004).

11 Linsley, B.K., Zhang, P., Kaplan, A., Howe, S. S. & Wellington, G. M. Decadal-Interdecadal Climate Variability from Multi-Coral Oxygen Isotope Records in the South Pacific Convergence Zone Region Since 1650AD, *Paleoceanography,* vol 23, PA2219, doi:10.1029/2007PA001539, (2008).

12 Ren, L., Linsley, B. K., Wellington, G. M., Schrag, D. P. & Hoegh-Guldberg, O. Deconvolving the $\delta^{18}O_{seawater}$ Component from Subseasonal Coral $\delta^{18}O$ and Sr/Ca at Rarotonga in the Southwestern Subtropical Pacific for the period 1726-1997, *Geochimica et Cosmochimica, Acta,* vol 67, no. 9, 1609-1631, (2003).

13 Linsley, B. K., Kaplan, A., Gouriou, Y., Salinger, J., de Menocal, P. B., Wellington, G. M. & Howe, S. S. Tracking the extent of the South Pacific Convergence Zone since the early 1600s, *Geochem. Geophys. Geosyst.,* 7, Q05003, doi:10.1029/2005GC001115 (2006).

14 Linsley, B. K., Dunbar, R. B., Lee, D., Tangri, N. & Dassié, E. P. Abrupt Northward Shift of SPCZ position in the late-1920s Indicates Coordinated Atlantic and Pacific ITCZ Change, *Past Global Changes Magazine*, Volume 25, No. 1, *CLIVAR Exchanges* No. 72, June 2017, doi:10.22498/pages.25.1.52. (2017).

15 Tangri, N., Dunbar, R. B., Linsley, B. K. & Mucciarone, D. M. ENSO's shrinking twentieth-century footprint revealed in a half-millennium coral core from the South Pacific Convergence Zone. *Paleoceanography and Paleoclimatology*, 33, 1136-1150, doi:10.1029/2017PA003310 (2018).

16 Stolorow, A. Assessing the paleoceanographic potential of the coral *Astreapora myriophthalma* at Fanning Atoll, central equatorial Pacific, M.Sc thesis, 110pp., University at Albany SUNY (2006).

17 Linsley, B.K., Dunbar, R. B., Wellington, G. M. & Mucciarone, D. A. A coral based reconstruction of Intertropical Convergence Zone variability over Central America since 1707, *Journal of Geophysical Research*, vol. 99, no. C5, 9977-9994 (1994).

18 Brenner, L. D., Linsley, B. K. & Potts, D. C. A modern Sr/Ca-δ^{18}O-Sea Surface Temperature calibration for *Isopora* corals on the Great Barrier Reef, *Paleoceanography,* 32. *doi:*10.1002/2016PA002973 *(2017).*

19 Brenner, L.D., Linsley, B. K. & Dunbar, R. B. Examining the utility of coral Ba/Ca as a proxy for river discharge and hydroclimate variability at Coiba Island, Gulf of Chiriquí, *Marine Pollution Bulletin*, doi:10.1016/j.marpolbul.2017.02.013 (2017).

20 Lemley, G. Assessing δ^{18}O in the coral genus *Isopora* for reconstructing Indo-Pacific regional and seasonal climate variability, M. Sc. Thesis, 63pp. University at Albany-SUNY (2012).

21 Quinn, T.M., Taylor, F.W. & Crowley, T.J. Coral-based climate variability in the Western Pacific Warm Pool since 1867. *Journal of Geophysical Research: Oceans*, 111, doi:10.1029/2005JC003243 (2006).

22 Evans, M.N., Fairbanks, R. G., & Rubenstone, J. L. The thermal oceanographic signal of El Niño reconstructed from a Kiritimati Island coral, *J. Geophys. Res*., 104 (C6), 13409-13421, (1999).

23 Quinn, T.M., Crowley, T.J., Taylor, F.W., Henin, C., Joannot, P. & Join, Y. A multicentury stable isotope record from a New Caledonia coral: interannual and decadal sea surface temperature variability in the southwest Pacific since 1657 A.D. *Paleoceanography* 13 (4), 412, doi: 10.1029/98PA00401 (1998).

24 Guilderson, T. P. & Schrag, D. P. Reliability of coral isotope records from the western Pacific warm pool: A comparison using age optimized records, *Paleoceanography,* 14(4), 457–464, doi:10.1029/1999PA900024 (1999).

25 Druffel, E.R.M. & Griffin, S. Large variations of surface ocean radiocarbon: evidence of circulation changes in the southwestern Pacific. *J. Geophys. Res*., 98(c11), 20246-20259 (1993).

26 Druffel, E.R.M. & Griffin, S. Variability of surface ocean radiocarbon and stable isotopes in the southwestern Pacific. *J. Geophys. Res*., - Ocean, 104 (C10), 23,607-613 (1999).

27 Moses, C.S. & Swart, P. K. Stable isotope and growth records in corals from the island of Tobago: Not simply a record of the Orinoco. *Proceedings of the 10th International Coral Reef Symposium* (Okinawa): 580-587 (2006)

28 Kuhnert H, Pätzold J, Hatcher B, Wyrwoll K-H, Eisenhauer A, Collins LB, Zhu Z. R. & Wefer G. A 200-year coral stable oxygen isotope record from a high-latitude reef off Western Australia. *Coral Reefs,* 18, 1-12 (1999).

29 Hess, G.A. Coral reefs in the Red Sea: Growth, production and stable isotopes. GEOMAR Report, 32:1-141 (1994)

30 Zinke, J., Pfeiffer, M., Timm, O., Dullo, W.-Ch. & Brummer, G. J. A. Western Indian Ocean marine and terrestrial records of climate variability: a review and new concepts on land-ocean interactions since AD 1660. *International Journal of Earth Sciences*, Vol. 98, 115–133, doi:10.1007/s00531-008-0365-5 (2009).

31 Zinke, J., Dullo, W.C., Heiss, G.A. & Eisenhauer, A. ENSO and Indian Ocean subtropical dipole variability is recorded in a coral record off southwest Madagascar for the period 1659 to 1995. *Earth and Planetary Science Letters*, 228, 177-194, doi: 10.1016/j.epsl.2004.09.028 (2004).

32 Boiseau, M.; Ghil, M. & Juillet-Leclerc, A. Climatic trends and interdecadal variability from South-Central Pacific coral records. *Geophysical Research Letters*, 26 (1999).

33 Felis, T., Suzuki, A., Kuhnert, H., Dima, M., Lohmann, G. & Kawahata, H. Subtropical coral reveals abrupt early-twentieth-century freshening in the western North Pacific Ocean. *Geology*, 37(6), 527-530, doi:10.1130/G25581A.1 (2009).

34 Osborne, M.S, Dunbar, R.B., Mucciarone, D.A., Druffel, E. & Sanchez-Cabeza. J.-A. A 215-yr coral $\delta^{18}O$ time series from Palau records dynamics of the West Pacific Warm Pool following the end of the Little Ice Age. *Coral Reefs*, doi:10.1007/s00338-014-1146-1 (2014).

35 Kilbourne, K.H.; Quinn, T.M.; Taylor, F.W.; Delcroix, T. & Gouriou, Y. El Niño-Southern Oscillation-related salinity variations recorded in the skeletal geochemistry of a *Porites* coral from Espiritu Santo, Vanuatu. *Paleoceanography*, 19. doi:10.1029/2004PA001033 (2004).

36 Quinn, T. M., Taylor, F. M. & Crowley, T. G. A 173 year stable isotope record from a tropical South Pacific coral, *Quat. Sci. Rev.,* 12, 407-418 (1993).

37 Swart, P.K., Dodge, R. E. & Hudson, H. J. A 240-year stable oxygen and carbon isotopic record in a coral from South Florida: Implications for the prediction of precipitation in southern Florida. *Palaios* 11(4): 362-375 (1996).

38 Kuhnert, H., Crüger, T. & Pätzold, J. NAO signature in a Bermuda coral Sr/Ca record. *Geochemistry, Geophysics, Geosystems*, 6(4). doi:10.1029/2004GC000786 (2005).

39 Nozaki, Y. Rye, D. M. Turekian, K. K. &Dodge, R. E. A 200 year record of carbon-13 and carbon-14 in a Bermuda coral, *Geophys. Res. Lett.,* vol. 5, no. 10, 825-828 (1978).

40 Guilderson, T. P., Fairbanks, R G. & Rubenstone, J. L. Tropical Atlantic coral oxygen isotopes: glacial-interglacial sea surface temperatures and climate change, *Marine Geology* 172, 75-89 (2001).
